# High Choline Intake during Pregnancy Reduces Characteristics of the Metabolic Syndrome in Male Wistar Rat Offspring Fed a High Fat But Not a Normal Fat Post-Weaning Diet

**DOI:** 10.3390/nu13051438

**Published:** 2021-04-24

**Authors:** Rola Hammoud, Emanuela Pannia, Ruslan Kubant, Adam Metherel, Rebecca Simonian, Zdenka Pausova, G. Harvey Anderson

**Affiliations:** 1 Department of Nutritional Sciences, Faculty of Medicine, University of Toronto, Toronto, ON M5S 1A8, Canada; rola.hammoud@mail.utoronto.ca (R.H.); e.pannia@mail.utoronto.ca (E.P.); ruslan.kubant@utoronto.ca (R.K.); adam.metherel@utoronto.ca (A.M.); rebecca.simonian@mail.utoronto.ca (R.S.); zdenka.pausova@sickkids.ca (Z.P.); 2 Department of Physiology, Faculty of Medicine, University of Toronto, Toronto, ON M5S 1A8, Canada; 3 The Hospital for Sick Children, Toronto, ON M5G 1X8, Canada

**Keywords:** choline, maternal nutrition, high fat diet, pregnancy, metabolic syndrome, fatty acids, liver

## Abstract

Maternal choline intakes are below recommendations, potentially impairing the child’s later-life metabolic health. This study aims to elucidate the interaction between the choline content of the gestational diet (GD) and fat content of the post-weaning diet (PWD) on metabolic phenotype of male Wistar rats. Pregnant Wistar rats were fed a standard rodent diet (AIN-93G) with either recommended choline (RC, 1 g/kg diet choline bitartrate) or high choline (HC, 2.5-fold). Male pups were weaned to either a normal (16%) fat (NF) or a high (45%) fat (HF) diet for 17 weeks. Body weight, visceral adiposity, food intake, energy expenditure, plasma hormones, triglycerides, and hepatic fatty acids were measured. HC-HF offspring had 7% lower body weight but not food intake, and lower adiposity, plasma triglycerides, and insulin resistance compared to RC-HF. They also had increased hepatic *n*-3 fatty acids and a reduced *n*-6/*n*-3 and C 18:1 *n*-9/C18:0 ratios. In contrast, HC-NF offspring had 6–8% higher cumulative food intake and body weight, as well as increased leptin and elevated hepatic C16:1 *n*-7/C16:0 ratio compared to RC-NF. Therefore, gestational choline supplementation associated with improved long-term regulation of several biomarkers of the metabolic syndrome in male Wistar rat offspring fed a HF, but not a NF, PWD.

## 1. Introduction

Maternal nutritional imbalances during pregnancy may alter metabolic adaptive responses in the fetus in utero, resulting in long-term metabolic changes to prepare the newborn for the post-natal environment [1]. One possible mechanism facilitating these early-life adaptations involve DNA-methylation-dependent epigenetic modifications affecting gene expression associated with the regulation of energy balance and metabolism [2,3].

Choline is an important nutrient, contributing to methylation reactions as a precursor to betaine, and may also be converted to several phospholipids that are essential for proper fetal development as well as the regulation of liver lipid transport and metabolism [4]. Despite being an essential nutrient during gestation, epidemiological studies show that less than 10% of North American pregnant women meet the adequate dietary intake requirements for choline [5]. It is also absent from most multivitamin prenatal supplements [6], raising concern of possible adverse health effects. We recently showed that a high (2.5-fold) choline diet consumed by Wistar rats during pregnancy programs long-term hypothalamic energy regulation in their offspring [7]. Higher expression of the orexigenic neuropeptide-Y neurons in newborn pups reflected their later-life increase in cumulative food intake and body weight gain when fed a normal fat (NF, 16% of calories) post-weaning diet (PWD) [7]. 

Because the North American diet averages around 36% fat [8], investigating the effect of choline supplementation during pregnancy on the offspring’s long-term metabolic response to a HF diet is timely and relevant. One recent report in mice identified a protective effect of choline when increased 4.5-fold in a 60% fat diet fed to mice 4 weeks before mating and throughout pregnancy on the development of obesity in the offspring maintained on a 60% fat diet [9]. However, the potential protective effect of moderate physiologically relevant choline intakes (2.5-fold) only during pregnancy on the offspring’s long-term metabolic response to a HF PWD has not been fully explored. We hypothesized that addition of choline to a NF diet consumed during pregnancy reduces characteristics of the metabolic syndrome in the offspring fed a HF diet. Pregnant Wistar rats were fed an AIN-93G diet with either the recommended choline or a 2.5-fold increase. Male pups were weaned to either a diet with 16% or 45% fat calories for 17 weeks. Body weight, food intake, visceral adiposity, plasma glucoregulatory hormones and triglycerides, and plasma and hepatic fatty acids were measured.

## 2. Materials and Methods

### 2.1. Animals and Diets

This experiment has been approved by the Animal Care Committee in the University of Toronto. First-time, 2-days pregnant Wistar rats were received from Charles River (Montreal, QC, Canada) and housed individually in plastic ventilated cages with food and water provided ad-libitum. The animals were kept on a 12:12 h light-dark cycle with lights on at 7 am and room temperature maintained at 22 ± 1 °C. Dams were randomized by weight upon arrival into two dietary treatment groups (*n* = 12–14/group) fed the standard NF (7% wt/wt, 16% Kcal) AIN-93G diet (D10012; Research Diets, New Brunswick, NJ, USA) with either recommended choline (RC) (1 g/kg diet choline from choline bitartrate) or high choline (HC, 2.5-fold recommended) only during pregnancy. Details on the selected choline amounts used in the gestational diet (GD) has been published previously [7]. Briefly, the amount of choline added was calculated according to the FDA dose conversion from humans to animals [10]. The HC GD contained 2.5 g of free choline which approximates to 1.1 g of choline per 1800 Kcal consumed which is below the tolerable upper limit (3.5 g per day). Each litter was sexed and culled to 6 pups per dam within 24 h of birth. Pups were terminated by rapid decapitation and trunk blood was collected from 1 male pup per dam and immediately frozen on dry ice and stored at −80 °C for later plasma leptin and insulin measures at birth. All dams were switched to the D10012G diet during the 3-week lactation period. At weaning, all dams were sacrificed and two male offspring per dam were weaned to either the NF diet (D10012G) or a HF diet (24% wt/wt, 45% Kcal) (D07101303R, Research Diets, New Brunswick, NJ, USA) for 17 weeks post-weaning (Tables S1 and S2). The dietary choline content of the post-weaning diets was adjusted to provide a similar amount of choline from the HF and NF diets (0.278 vs. 0.297 mmol/day). During the entire experimental period, food intake was measured weekly for offspring and dams. Body weight of dams was measured on arrival and at birth. Offspring body weight was measured at birth and weekly from weaning to 17 weeks post-weaning. At 17 weeks post-weaning and after a 6-h daytime fast, the adult male offspring were terminated by rapid decapitation for trunk blood and liver tissue collection. Visceral fat pads (retroperitoneal, mesenteric, and epididymal) were dissected and weighed, and the visceral adiposity index was calculated from the total visceral fat-pad mass (g) as a percent of final body weight. All tissue weights are expressed as a percentage (%) of final body weight (fbw). 

### 2.2. Plasma Glucoregulatory Hormones, Total Free Fatty Acids, and Triglycerides

Fasting plasma was analyzed in male offspring using commercial ELISAs for leptin (Cat. no. EZRL-83K; EMD Millipore, Billerica, MA, USA) and insulin (Cat. no. 80-INSRT-E01, ALPCO, New Hampshire, USA) at birth and 17 weeks post-weaning. Post-weaning plasma leptin concentrations were adjusted for total visceral fat-pad mass (g). Fasting plasma glucose (Cat#10009582, Cayman Chemical Co., Ann Arbor, MI, USA) was measured at 17 weeks post-weaning, and the homeostatic model assessment of insulin resistance (HOMA-IR) was calculated as:

fasting plasma glucose (mg/dL) × fasting plasma insulin (μU/mL)/2430(1)

Post-weaning total plasma free fatty acids (FFA, Cat# ab65341, Abcam) and plasma triglycerides (Cat#10010303, Cayman Chemical, Ann Arbor, MI, USA) were also measured according to the manufacturers’ instructions.

### 2.3. Energy Expenditure and Activity

A subset of rat offspring (*n* = 8/group) was transferred to SickKids Hospital (Toronto, ON, Canada) for the measurement of the whole-body energy expenditure. Animals were placed in the Comprehensive Laboratory Animal Monitoring System (CLAMS; Columbus Instruments, Columbus, OH, USA) at week 15 post-weaning as previously described [7]. Briefly, rats were individually housed (12 h light-dark cycle; light was turned on from 6 a.m. to 6 p.m.) and measurements taken at 23 °C room temperature. Each rat was monitored for two uninterrupted 24-h periods, to allow for acclimatization in the first 24-h and data collection and analysis was performed during the second 24-h period as described [11,12]. The input air for the chambers and the expired air were sequentially analyzed for CO_2_ and O_2_ with a gas analyzer every 13 min. Chamber air flow rate was adjusted to 2 liters per minute and an extracted outflow to 0.4 liters per minute. Whole-body energy expenditure was estimated as heat production, automatically calculated from O_2_ consumption (VO_2_) and CO_2_ production (VCO_2_) as follows:

heat (kcal/kg/h) = (3.815 + 1.232 × (VCO_2_/VO_2_)) × VO_2_ (mL/kg/h) (2)

The respiratory exchange ratio (RER) was automatically derived from VCO2/VO2. Animal activity was measured using the Opto-M3 activity monitor. Beam interruptions along the *x*-axis were scored for ambulation (XAMB) and total beam interruptions were scored as total locomotor activity along the *x*-axis (XTOT). Stereotypy or rearing activity (ZTOT) was automatically derived as number of vertical or *z*-axis beam interruptions.

### 2.4. Liver Lipid Extraction and Fatty Acid Quantification

Liver tissue was collected from adult offspring (*n* = 10/group) at 17 weeks post-weaning and total lipids were extracted according to the Folch et al. method [13]. Briefly, 100 mg of liver tissue was treated with 2:1 chloroform: methanol and triheptadecanoic acid (17:0) was added as an internal standard to quantify fatty acids. The tissue was then homogenized and left to incubate at 4 °C overnight. Samples were then treated with 0.88% KCl and phase separation was achieved by a 10-min centrifugation at 500× *g*. The lipid-containing organic phase was collected and dried down under nitrogen and reconstituted in chloroform. Fatty acid methyl esters were generated and quantified as previously described [14], by which boron trifluoride/methanol (14%; Sigma-Aldrich, St. Louis, MO, USA) was added to the extracted lipids and heated at 100 °C for 60 min. Fatty acid methyl esters were analyzed by a gas chromatograph (model 430; Varian) equipped with a 30-m DB-FFAP column. The injector and flame-ionization detector were set at 250 °C and 300 °C, respectively, and the oven-temperature program was 50 °C for 1 min, increased to 130 °C at 30 °C/min, then increased to 175 °C at 10 °C/min, then increased to 230 °C at 5 °C/min and held for 9.5 min, and finally increased to 240 °C at 50 °C/min and held for 11.1 min. Helium was the carrier gas and was set to a constant flow rate of 1.0 mL/min. Fatty acids were identified using the Compass CDS chromatography software, and concentrations were calculated as ug fatty acid per gram of liver tissue as previously described [14].

### 2.5. Statistical Analysis

This study investigating the offspring’s metabolic response to a choline GD when fed a NF or HF PWD and includes a follow-up analysis using a subset of previously unpublished data (HC-HF and RC-HF groups) compared against a subset of data (RC-NF and HC-NF) from a recently published study [7]. However, all GD and PWD interventions and plasma and tissue measures in the current study were performed simultaneously, therefore there are no confounding batch differences to report. All data were analyzed using SAS Version 9.4 software (SAS Institute Inc., Carey, NC, USA). A two-way analysis of variance (ANOVA) was performed using PROC GLIMMIX procedure with GD and PWD as main factors and a GD×PWD interaction term for all measures. All significant interactions were followed by a planned comparison using Student’s T-test assessing differences between groups stratified by PWD (RC-NF vs. HC-NF) and (RC-HF vs. HC-HF). To assess the relationship between cumulative food intake and final body weight within groups, a Pearson correlation analysis was performed using PROC CORR procedure. On all occasions, statistical significance was declared at *p*-value ≤ 0.05. All values are expressed as mean ± SEM.

## 3. Results

### 3.1. Litter Size, Body Weight, and Plasma Leptin and Insulin Concentrations of Pups at Birth

The choline content of the GD had no effect on the litter size and average body weights of pups at birth. Plasma insulin concentrations were not affected by maternal diet. However, male pups born from dams fed an HC diet during pregnancy had significantly lower plasma leptin concentration (ng/mL) compared to RC (control) group (Table S3).

### 3.2. Cumulative Food Intake and Final Body Weight of Adult Male Offspring at 17 Weeks Post-Weaning

**Figure 1:**
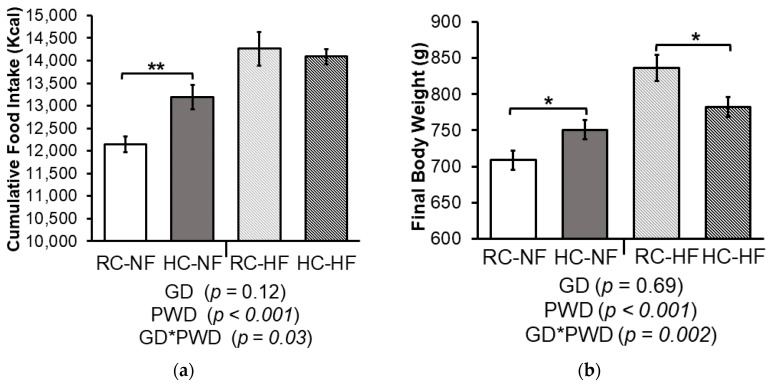
(**a**) Cumulative food intake (Kcal) and (**b**) final body weight (g) of male offspring born to dams fed either a recommended choline (RC) or a high choline (HC) gestational diet (GD) then weaned to either a normal fat (NF) or high fat (HF) post-weaning diet (PWD) for 17 weeks. Values are mean ± SEM, *n* = 10–12/group. Data were analyzed using a 2-way ANOVA to test the interaction effect of GD and PWD. * *p* < 0.05 ** *p* < 0.01 by Student’s T-test comparing groups stratified by PWD. Abbreviations: RC-NF: recommended choline (1 g/kg) gestational diet weaned to normal fat (16%kcal) post-weaning diet; HC-NF: high choline (2.5 g/kg) gestational diet weaned to normal fat (16% Kcal) post-weaning diet; RC-HF: recommended choline (1 g/kg) gestational diet weaned to high fat (45%Kcal) post-weaning diet; HC-HF: high choline (2.5 g/kg) gestational diet weaned to high fat (45%Kcal) post-weaning diet.

As anticipated, there was a PWD effect on cumulative food intake (Kcal). Adult offspring maintained on a HF diet consumed more calories over the 17-week post-weaning period compared to those fed the NF diet. (Figure 1a). However, there was a significant interaction effect between the choline content of the GD and the fat content of the PWD on cumulative food intake (P _GD*PWD_ = 0.03). When stratified by PWD, HC-NF offspring had 8% higher food intake compared to RC-NF (*p* < 0.01), but cumulative food intake was not different between RC-HF and HC-HF offspring. Similarly, there was an interaction between GD and PWD on final body weight (P _GD*PWD_ = 0.002). HC-NF offspring had 6% higher final body weight (*p* < 0.05) compared to RC-NF, whereas HC-HF offspring had 7% lower final body weight compared to RC-HF (*p* < 0.05) (Figure 1b). Pearson correlation analysis showed that the final body weights of RC-NF, HC-NF, and RC-HF offspring were strongly and significantly positively correlated with cumulative food intake (RC-NF r = 0.84, *p* = 0.001, HC-NF r = 0.90, *p* = 0.0001, RCHF r = 0.62, *p* = 0.038). Thus, food intake in these offspring was a primary contributor to their final body weight. In contrast, the relationship in the HC-HF offspring was not significant (r = 0.19, *p* = 0.55). This observation suggests that the HC gestational diet programmed determinants of their healthier body composition and metabolic phenotype by mechanisms independent of food intake. The choline content of the GD had no effect on weekly food intake (g) of dams during pregnancy and lactation and thus did not confound the offspring’s food intake data (Table S4).

### 3.3. Energy Expenditure and Activity of Adult Male Offspring at 15 Weeks Post-Weaning

**Figure 2:**
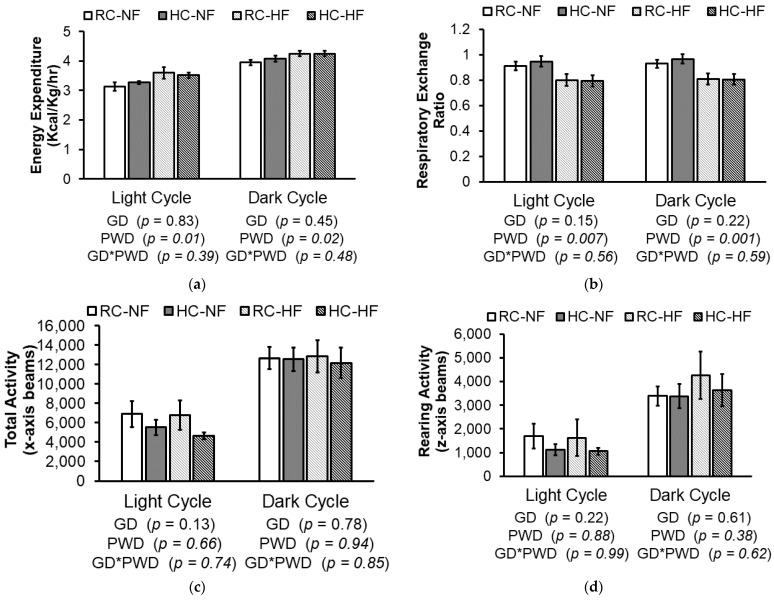
(**a**) Average energy expenditure measured as heat decimated in Kcal/kg/hr, (**b**) average respiratory exchange ratio, (**c**) total locomotor activity (XTOT, ambulation and stereotypy) along the *x*-axis (number of beam breaks), (**d**) and rearing activity along the *z*-axis (ZTOT, number of beam breaks) measured over a 24-h period at 15 weeks post-weaning in male offspring born to dams fed either a recommended choline (RC) or a high choline (HC) gestational diet (GD) then weaned to either a normal fat (NF) or high fat (HF) post-weaning diet (PWD). Values are mean ± SEM, *n* = 6–7/group. Data were analyzed using a 2-way ANOVA to test the main effects of the GD and PWD as well as their interaction. Abbreviations: RC-NF: recommended choline (1 g/kg) gestational diet weaned to normal fat (16%kcal) post-weaning diet; HC-NF: high choline (2.5 g/kg) gestational diet weaned to normal fat (16% Kcal) post-weaning diet; RC-HF: recommended choline (1 g/kg) gestational diet weaned to high fat (45%Kcal) post-weaning diet; HC-HF: high choline (2.5 g/kg) gestational diet weaned to high fat (45%Kcal) post-weaning diet.

Average energy expenditure measured as heat decimated in Kcal per Kg of body weight per hour over 24 h, was higher in rats fed the HF PWD during both the light cycle (*P _PWD_* = 0.01) and dark cycle (*P _PWD_* = 0.02), but there were no significant GD or GD*PWD interaction effects (Figure 2a). Similarly, the respiratory exchange ratio (RER) during the dark and light cycles directly reflected the offspring’s PWD by which the HF groups had significantly lower RER compared to the NF groups (Figure 2b), demonstrating a higher utilization of lipids as an energy source by the HF offspring compared to NF. Finally, there was no significant main effect of GD or PWD nor an interaction effect on ambulation and total locomotor activity (XTOT) along the *x*-axis, as well as rearing activity along the *z*-axis (ZTOT) (Figure 2c,d). Therefore, these results suggest that the observed interaction effects of the GD with PWD on food intake and final body weight of the adult rat offspring may not be related to alteration in their energy expenditure and activity levels later-in-life. However, further research examining changes at an earlier stage of development may provide additional insight on the factors contributing to the later-life phenotypic outcomes.

### 3.4. Liver Weight, Visceral Adiposity, and Fasting Plasma Metabolic Measures of Adult Male Offspring at 17 Weeks Post-Weaning

Visceral adiposity measured as percent final body weight (%fbw) was higher in the HF groups compared to those fed the NF PWD (*P _PWD_* <0.001) but was dependent on the GD choline content (P _GD*PWD_ = 0.03). The HC-HF offspring had 15% lower visceral adiposity (*p* < 0.05) compared to RC-HF, whereas visceral adiposity in HC-NF offspring was not different from RC-NF (*p* > 0.05) (Table 1). Similarly, there was a GD and PWD interaction effect on fasting plasma leptin concentrations both before and after normalizing for visceral adiposity (P _GD*PWD_ < 0.05), as well on fasting plasma insulin (P _GD*PWD_ = 0.006), HOMA-IR (P _GD*PWD_ = 0.04), and plasma triglycerides (P _GD*PWD_ = 0.04) (Table 1). When stratified by PWD, HC-HF offspring had 18% lower insulin (*p* < 0.05), 24% lower HOMA-IR (*p* < 0.01), and 30% lower plasma triglycerides (*p* < 0.05) but no difference in plasma leptin concentrations compared to RC-HF. In contrast, HC-NF offspring had 22% higher plasma leptin adjusted for adiposity (*p* < 0.05) compared to RC-NF offspring, but similar plasma insulin concentrations (*p* > 0.05), HOMA-IR (*p* > 0.05), and plasma triglycerides (*p >* 0.05) compared to RC-NF offspring (Table 1). Liver weights (% fbw) and fasting plasma total FFA concentrations in male Wistar rat offspring at 17 weeks post-weaning were not different between groups (Table 1).

### 3.5. Hepatic Fatty Acid Composition of Adult Male Offspring at 17 Weeks Post-Weaning

All offspring weaned to a HF PWD had ~30% higher total hepatic fatty acids compared to those weaned to a NF diet (*P _PWD_* = 0.001) (Table 2). The HF groups also had higher total saturated fatty acid (SFAs), monounsaturated fatty acids (MUFAs), omega 6 fatty acids (*n*-6), and polyunsaturated fatty acids (PUFAs) (Table 2). Total omega-3 fatty acids (*n*-3) were also higher in the HF groups compared to NF groups (P *_PWD_* = 0.013) but this effect was dependent on the choline content of the GD as indicated by a significant interaction effect (P _GD*PWD_ = 0.04) (Table 2). When stratified by PWD, HC-HF offspring had higher total *n*-3 compared to RC-HF (~33%, *p* <0.05), whereas hepatic *n*-3 concentrations were not different between NF groups in response to the gestational choline diet (Table 2). A main effect of the PWD on individual fatty acid concentrations (µg/g) was observed in the liver. The HF groups had higher concentrations of several SFAs (i.e., C16:0, C20:0, C22:0, and C24:0), MUFAs (i.e., C18:1 *n*-7, C18:1 *n*-9, and C20:1 *n*-9), and PUFAs (i.e., C18:2 *n*-6, C18:3 *n*-6, C20:2 *n*-6, C22:2 *n*-6, C22:4n-6, C22:5 *n*-6, and C18:3 *n*-3) (Table 3). However, there was a significant interaction effect between the GD and PWD on C18:0, C16:1 *n*-7, C20:4 *n*-6, and C22:6 *n*-3 concentrations (Table 3). HC-HF offspring had ~38% higher C18:0 compared to RC-HF (*p* < 0.05), but no differences were seen in C18:0 concentrations between NF groups. HC-HF offspring also had higher C20:4 *n*-6, and C22:6 *n*-3 concentrations compared to RC-HF (*p < 0.05*) but were similar between NF groups. Furthermore, C16:1 *n*-7 was higher in HC-NF compared to RC-NF groups (*p* < 0.01) but was not different between HF groups (Table 3). Several fatty acid ratios known to be of metabolic significance were affected by the choline content of the maternal diet (Table 4). There was an interaction between the choline content of the GD and the fat content of the PWD on the C18:1n9/C18:0 and *n*-6/*n*-3 ratios (P _GD*PWD_ < 0.05) (Table 4). Offspring born from dams fed the HC GD had lower C18:1 *n*-9/C18:0 and *n*-6/*n*-3 ratios compared to those born from RC dams only when weaned to a HF diet for 17 weeks. There were no GD or PWD main effects nor an interaction effect on the docosahexaenoic acid (DHA) to arachidonic acid (ARA) ratio (DHA/ARA) and DHA to eicosapentaenoic acid (EPA) ratio (DHA/EPA) between groups. However, further analysis by Student’s t-test stratified by PWD showed that the HC-HF offspring had moderately elevated DHA/ARA (*p* = 0.047) and DHA/EPA (*p* = 0.065) compared to RC-HF (Table 4).

## 4. Discussion

The results of this study support our hypothesis that an increase in choline in a NF diet consumed during pregnancy reduces characteristics of the metabolic syndrome in the offspring fed a HF PWD. Choline supplementation during pregnancy had opposing effects on the offspring’s long-term metabolic phenotype when fed a HF vs. NF PWD for 17 weeks. When fed a HF PWD, male offspring from the HC-fed dams had no difference in food intake, but lower final body weight associated with lower visceral adiposity, lower fasting plasma insulin, and improved glucose regulation compared to those born from RC dams. This suggests that the gestational choline diet may have programmed the offspring toward improved later-life body composition and metabolic phenotype compared to RC-HF, independent of changes in food intake regulation. HC-HF offspring also had lower plasma triglycerides, higher hepatic *n*-3 PUFA and DHA, and lower *n*-6/*n*-3 and C18:1 *n*-9/C18:0 ratios compared to RC-HF offspring. In contrast, offspring fed a NF PWD and born to dams fed the HC diet had higher cumulative food intake and final body weight as well as increased leptin and elevated hepatic C16:1 *n*-7/C16:0 ratio compared to controls.

These results are consistent with those showing a protective effect of choline supplementation (4.5-fold) to a HF diet prior to conception and throughout gestation in mice on the offspring’s metabolic phenotype [9,15]. However, they contrast with our previous studies exploring other methyl vitamins, showing that a diet containing 10-fold vitamins B6, B12, and folic acid during pregnancy associated with higher food intake and body weight in male offspring fed a HF PWD diet [16]. These effects have been attributed to the methyl-group vitamin folic acid [17] and have shown that a high folic acid GD resulted in obesity and the metabolic syndrome in male Wistar rat offspring exposed to either a standard [18] or a HF PWD, with the obesogenic phenotype exacerbated in the latter [19]. These studies also showed an interaction effect between choline and folic acid in the GD on the programming of the metabolic phenotype of the offspring. Adding choline to the high folic acid maternal diets reduced the folic acid-dependent increase in the offspring’s later-life food intake and body weight-gain [20].

Furthermore, this study provides evidence that the interaction between maternal choline intakes with the fat composition of the PWD programs the regulation of food intake and metabolism in the male offspring. When maintained on a NF PWD for 17 weeks, offspring born to dams fed the HC pregnancy diets had increased cumulative food intake and final body weight, but similar energy expenditure and total activity compared to RC offspring, suggesting that the increased body weight gain could be mainly attributed to later-life changes in food intake regulation. At birth, pups born to dams fed the HC diet during pregnancy had lower plasma leptin concentrations compared to those born to RC dams. HC-NF adult offspring had increased leptin concentrations suggesting possible later-life compensatory mechanism to restore the leptin balance altered at birth. Leptin exerts its central effects through the hypothalamus in part by suppressing the signaling of orexigenic neuronal populations [21]. We have previously reported that HC newborn pups had high expression of the orexigenic NPY neuron [7] and associate with their higher body weight and food intake when fed a NF PWD. In contrast, as reported in this study, HC offspring fed a HF PWD had lower body weight compared to RC-HF offspring. These results show differences in the metabolic response of the offspring due to the PWD. Notably, leptin continues to drive the development of hypothalamic regulatory pathways up to four weeks post-birth [22] and its secretion is under epigenetic control [23,24], thus providing plausibility that the postnatal diet may have interacted with the GD and continued to modulate its signaling and response. In addition to effects on central energy regulation, leptin also exerts effects peripherally and has been shown to reduce hepatic lipogenesis and lipid accumulation [25]. Consistent with the potential leptin-resistant phenotype observed in HC-NF compared to RC-NF offspring, dysregulation in liver fatty acid metabolism was indicated. HC-NF offspring had increased palmitoleic acid (C16:1 *n*-7) to palmitic acid (C16:0) ratio, a marker of stearoyl-CoA desaturase 1 (SCD-1) activity, compared to RC-NF. A previous study showed that *ob/ob* mice that do not express leptin, have upregulated *scd-1* mRNA expression and enzyme activity for the conversion of hepatic C16:0 to C16:1 *n*-7 that is associated with increased hepatic lipid accumulation of which can be corrected with 12 days of leptin treatment [26]. Notably, a bidirectional effect is known where changes in hepatic fatty acid composition also regulate the production and signaling of leptin and as first reported herein, may be modulated by the choline content of the GD. Further research is needed to elucidate the mechanisms underlying the interaction between leptin and fatty acids observed in this study.

A prominent role for choline in the regulation of liver lipid metabolism has been clearly identified in adult rodent and human studies [27]. However, this is the first study to identify a long-term programming effect of maternal choline supplementation on liver fatty acid composition in adult rat offspring. Choline content of the maternal diet markedly affected hepatic fatty acid composition in mature offspring fed both a HF and a NF PWD even though they differed in fatty acid composition. HC-HF offspring had higher liver DHA, ARA, DHA/ARA and total *n*-3, and lower *n*-6/*n*-3 and C18:1n9/C18:0 compared to RC-HF, and associated with their lower fasting plasma triglycerides and insulin resistance as indicated by HOMA-IR [28]. HC-NF offspring had higher liver C16:1 *n*-7/16:0 compared to RC-NF groups. These results indicate that the effects of the gestational choline content of the diet elicits a different metabolic response on hepatic fatty acid metabolism dependent on the composition of the fat in the PWD. The GD and PWD both contained 7% wt/wt of soybean oil but lard was the predominant source in the HF PWD diet (13% wt/wt lard). Lard is a commonly used source of fat to induce obesity and the metabolic syndrome in animal models as it is stable and more effective compared to soybean oil [29]. The HF PWD contained ~43% more saturated fats from lard and ~30% higher *n*-6/*n*-3 ratio, and ~1.3-fold and 2-fold higher (C18:3 *n*-3) alpha *n*-3 linolenic and *n*-6 linoleic acid (C18:2 *n*-6) respectively compared to the standard AIN-93G diet (Supplementary Table S2). Despite these differences, our results highlight a possible mitigating effect of choline supplementation during pregnancy on hepatic lipid dysregulation in diet-induced obesity [30].

Several possible pathways by which gestational choline supplementation may have programmed beneficial effects on later-life hepatic lipid metabolism were identified. As both DHA and ARA were absent from the gestational diets, the increased concentrations of liver DHA and ARA observed in offspring born from dams fed HC gestational diets may suggest a programming effect of choline in utero on upregulating synthesis of these fatty acids, of which was later augmented by the HF PWD that contains higher concentrations of their dietary precursors *n*-3 alpha-linolenic acid (C18:3 *n*-3) and *n*-6 linoleic acid (C18:2 *n*-6) respectively [31]. However, a HF diet on its own has been shown to lower enzyme activity of the desaturase enzymes involved in ARA/DHA synthesis [32], and alternative explanations such as a slower metabolic consumption and/or higher incorporation into specific lipid species of DHA/ARA may exist. The gestational choline supplementation may have permanently altered choline utilization in the offspring resulting in an increase in phosphatidylcholine synthesis, a lipid species in which liver DHA is highly abundant, particularly during pregnancy [33]. DHA is required in abundance during pregnancy to support fetal growth and its synthesis and storage is increased during pregnancy in rats [14,34]. Furthermore, several reports in rodent and human studies have shown that combined maternal choline and DHA dietary intakes synergistically enhance DHA blood and tissue accretion by mothers and improves DHA placental transport and utilization by the developing fetus [35]. Additionally, pregnant women have greater blood phosphatidylcholine (PC)-DHA enrichment compared to non-pregnant women of child-bearing age [36], suggestive of greater activation of the phosphatidylethanolamine N-methyltransferase (PEMT) pathway during pregnancy and potential programming in the offspring [33,35]. Finally, the gestational choline diet affected the ratios of C18:1 *n*-9/18:0 and C16:1 *n*-7/16:0 which are indices of SCD-1 activity, a rate-limiting enzyme responsible for the conversion of SFAs into MUFAs [30,37,38,39,40], suggesting possible programming effects on this pathway and its interaction with the fat content of the PWD.

In summary, this study provides several lines of evidence that in utero programming of choline on the offspring’s metabolic phenotype is influenced by its amount in the GD, as well as the nutrient composition of both the GD and PWD. Choline supplementation during pregnancy led to programming of long-term food intake and hepatic lipid metabolism. Future research is needed to determine if these observed outcomes are due to epigenetic modifications of genes resulting from in utero exposure to the methyl donor choline.

A limitation of this study is the lack of follow-up measures to elucidate the potential mechanisms underlying the observed phenotypic effects in the offspring. Future research is needed to determine if these observed outcomes are due to transient or long-term tissue-specific alterations in choline and related 1-carbon metabolites. Moreover, this study did not explore sexual dimorphic differences in the offspring’s response to the GD and PWD, warranting future examination. However, a strength of this study is the use of physiologically relevant dietary interventions that may help inform future clinical research focused on enhancing maternal choline intakes. These findings encourage consideration of both the macro and micronutrient composition of the GD and PWD in determining the beneficial effects of the HC diet consumed during pregnancy on offspring health.

## 5. Conclusions

Gestational choline supplementation is associated with improved long-term regulation of several biomarkers of the metabolic syndrome in male Wistar rat offspring fed a HF, but not a NF, PWD.

## Figures and Tables

**Table 1:** Visceral adiposity and fasting plasma metabolic measures of adult male Wistar rat offspring ^1^.

	RC-NF	HC-NF	RC-HF	HC-HF	* P _GD_*	* P _PWD_*	P _GD*PWD_
Liver weight (% fbw)	2.6 ± 0.1	2.6 ± 0.1	2.5 ± 0.1	2.5 ±0.1	0.96	0.18	0.93
Visceral adiposity (% fbw)	6.5 ± 0.6	7.4 ± 0.5	11 ± 0.4	9.4 ± 0.5 *	0.6	<0.001	0.03
Leptin (ng/mL)	12.1 ± 1.21	16.5 ± 1.35 *	23.3 ± 1.69	21.6 ± 1.27	0.33	<0.001	0.03
Leptin/visceral adipose mass	0.25 ± 0.02	0.32 ± 0.02 *	0.3 ± 0.01	0.3 ± 0.02	0.04	0.4	* 0.04 *
Insulin (ng/mL)	2.08 ± 0.25	2.48 ± 0.12	2.97 ± 0.11	2.45 ± 0.14 *	0.7	0.009	0.006
Glucose (mg/dL)	128.7 ± 7.7	141.2 ± 11.3	139.7 ± 3.4	128.4 ± 8.4	0.9	0.8	0.15
HOMA-IR	3.1 ± 0.5	3.7 ± 0.4	4.2 ± 0.3	3.2 ± 0.2 **	0.5	0.4	0.04
FFA (mg/dL)	254 ± 16.1	252 ± 13.1	240 ± 8.43	228 ± 15.4	0.64	0.17	0.69
Triglycerides (nmol/dL)	90.8 ± 6.07	95.3 ± 6.51	89.6 ± 7.61	61.9 ± 8.59 *	0.12	0.02	0.04

^1^ Male Wistar offspring were born to dams fed either a recommended choline (RC) or a high choline (HC) gestational diet (GD) then weaned to either a normal fat (NF) or high fat (HF) post-weaning diet (PWD) for 17 weeks. Values are mean ± SEM, *n* = 8–11/group. Data were analyzed using a 2-way ANOVA to test the interaction effect of GD and PWD. * *p* < 0.05 ** *p* < 0.01 by Student’s *T*-test comparing groups stratified by PWD. Abbreviations: RC-NF: recommended choline (1 g/kg) gestational diet weaned to normal fat (16%Kcal) post-weaning diet; HC-NF: high choline (2.5 g/kg) gestational diet weaned to normal fat (16% Kcal) post-weaning diet; RC-HF: recommended choline (1 g/kg) gestational diet weaned to high fat (45%Kcal) post-weaning diet; HC-HF: high choline (2.5 g/kg) gestational diet weaned to high fat (45%Kcal) post-weaning diet; %fbw: percentage of final body weight, HOMA-IR: homeostatic model assessment of insulin resistance, FFA: free fatty Acids.

**Table 2:** Concentrations (µg/g) of total fatty acids in liver of adult male Wistar rat offspring ^1^.

Fatty Acids (µg/g)	RC-NF	HC-NF	RC-HF	HC-HF	*P _GD_*	*P _PWD_*	P _GD*PWD_
Ʃ SFAs	3.17 ± 0.26	3.53 ± 0.39	4.13 ± 0.45	4.99 ± 0.51	0.09	0.04	0.37
Ʃ MUFAs	1.51 ± 0.14	1.97 ± 0.22	3.42 ± 0.57	2.96 ± 0.40	0.78	0.001	0.40
Ʃ *n*-6 PUFA	4.78 ± 0.32	4.74 ± 0.58	6.57 ± 0.85	7.66 ± 0.54	0.26	0.001	0.24
Ʃ *n*-3 PUFA	1.13 ± 0.09	1.14 ± 0.16	1.19 ± 0.12	1.69 ± 0.06 *	0.04	0.013	0.04
Ʃ PUFAs	5.91 ± 0.41	5.88 ± 0.74	7.76 ± 0.97	9.35 ± 0.59	0.29	0.001	0.28
Total fatty acids	10.59 ± 0.76	11.37 ± 1.29	15.30 ± 1.93	17.31± 1.42	0.35	0.001	0.68

^1^ Male Wistar offspring were born to dams fed either a recommended choline (RC) or a high choline (HC) gestational diet (GD) then weaned to either a normal fat (NF) or high fat (HF) post-weaning diet (PWD) for 17 weeks. Values are mean ± SEM, *n* = 8–9/group. Data were analyzed using a 2-way ANOVA to test the interaction effect of GD and PWD. * *p* < 0.05 by Student’s T-test comparing groups stratified by PWD. Abbreviations: RC-NF: recommended choline (1g/kg) gestational diet weaned to normal fat (16%Kcal) post-weaning diet; HC-NF: high choline (2.5 g/kg) gestational diet weaned to normal fat (16% Kcal) post-weaning diet; RC-HF: recommended choline (1 g/kg) gestational diet weaned to high fat (45%Kcal) post-weaning diet; HC-HF: high choline (2.5 g/kg) gestational diet weaned to high fat (45%Kcal) post-weaning diet; Ʃ SFAs: total sum of saturated fatty acids; Ʃ MUFAs: total sum monounsaturated fatty acids; Ʃ *n*-6 PUFA: total sum of omega-6 polyunsaturated fatty acids; Ʃ *n*-3 PUFA: total sum of omega-3 polyunsaturated fatty acids; Ʃ PUFAs: total sum of all polyunsaturated fatty acids.

**Table 3:** Hepatic fatty acid composition of adult male Wistar rat offspring ^1^

Fatty Acids (µg/g)	RC-NF	HC-NF	RC-HF	HC-HF	*P _GD_*	*P _PWD_*	P_GD*PWD_
Myristic Acid (C14:0)	0.03 ± 0.01	0.04 ± 0.01	0.04 ± 0.01	0.04 ± 0.01	0.99	0.31	0.33
Palmitic Acid (C16:0)	1.71 ± 0.13	1.93 ± 0.19	2.59 ± 0.38	2.59 ± 0.26	0.68	0.008	0.68
Stearic Acid (C18:0)	1.39 ± 0.14	1.49 ± 0.19	1.64 ± 0.13	2.65 ± 0.08 *	0.006	0.001	0.003
Arachidic Acid (C20:0)	0.007 ± 0.001	0.009 ± 0.002	0.006 ± 0.001	0.006 ± 0.001	0.17	0.05	0.37
Behenic Acid (C22:0)	0.009 ± 0.001	0.012 ± 0.002	0.007 ± 0.001	0.008 ± 0.001	0.08	0.02	0.31
Tricosylic Acid (C23:0)	0.011 ± 0.003	0.018 ± 0.004	0.009 ± 0.002	0.014 ± 0.002	0.04	0.31	0.81
Lignoceric Acid (C24:0)	0.018 ± 0.002	0.023 ± 0.003	0.014 ± 0.002	0.018 ± 0.001	0.04	0.03	0.91
Palmitoleic Acid (C16:1 *n*-7)	0.15 ± 0.02	0.26 ± 0.04*	0.14 ± 0.03	0.11 ± 0.01	0.31	0.004	0.02
Vaccenic Acid (C18:1 *n*-7)	0.39 ± 0.04	0.51 ± 0.06	0.33 ± 0.05	0.35 ± 0.03	0.12	0.018	0.25
Oleic Acid (C18:1 *n*-9)	0.97 ± 0.09	1.19 ± 0.13	2.91 ± 0.49	2.48 ± 0.37	0.75	0.000	0.35
Eicosenoic Acid (C20:1 *n*-9)	0.01 ± 0.001	0.02 ± 0.002	0.04 ± 0.006	0.04 ± 0.004	0.46	0.000	0.81
Linoleic Acid (C18:2 *n*-6)	2.13 ± 0.16	1.96 ± 0.22	3.63 ± 0.64	3.48 ± 0.48	0.73	0.003	0.97
Gamma Linolenic Acid (C18:3 *n*-6)	0.03± 0.001	0.02 ± 0.001	0.05 ± 0.01	0.06 ± 0.01	0.91	0.000	0.62
Eicosadienoic Acid (C20:2 *n*-6)	0.04 ± 0.01	0.04 ± 0.01	0.05 ± 0.01	0.06 ± 0.01	0.55	0.017	0.58
Arachidonic Acid (ARA, C20:4 *n*-6)	2.37 ± 0.21	2.51 ± 0.35	2.49 ± 0.24	3.63 ± 0.13 *	0.013	0.014	0.05
Docosadienoic Acid (C22:2 *n*-6)	0.08 ± 0.02	0.08 ± 0.02	0.09 ± 0.02	0.16 ± 0.03	0.15	0.050	0.13
Adrenic Acid (C22:4 *n*-6)	0.05 ± 0.003	0.05 ± 0.007	0.12 ± 0.02	0.13 ± 0.02	0.91	0.000	0.55
Docosapentaenoic Acid (C22:5 *n*-6)	0.02 ± 0.002	0.02 ± 0.005	0.04 ± 0.006	0.05 ± 0.004	0.65	0.000	0.26
Alpha-linolenic Acid (ALA, C18:3 *n*-3)	0.095 ± 0.013	0.077 ± 0.010	0.170 ± 0.038	0.146 ± 0.028	0.43	0.012	0.92
Eicosatrienoic Acid (ETE, C20:3 *n*-3)	0.017 ± 0.009	0.004 ± 0.001	0.006 ± 0.001	0.006 ± 0.001	0.15	0.28	0.13
Eicosapentaenoic Acid (EPA, C20:5 *n*-3)	0.042 ± 0.005	0.042 ± 0.007	0.050 ± 0.011	0.050 ± 0.009	0.96	0.36	0.96
Docosapentaenoic Acid (DPA, C22:5 *n*-3)	0.11 ± 0.009	0.12 ± 0.03	0.13 ± 0.02	0.17 ± 0.02	0.19	0.11	0.61
Docosahexaenoic Acid (DHA, C22:6 *n*-3)	0.87 ± 0.09	0.89 ± 0.13	0.83 ± 0.08	1.32 ± 0.06 *	0.008	0.04	0.02

^1^ Male Wistar offspring were born to dams fed either a recommended choline (RC) or a high choline (HC) gestational diet (GD) then weaned to either a normal fat (NF) or high fat (HF) post-weaning diet (PWD) for 17 weeks. Values are mean ± SEM, *n* = 8–9/group. Data were analyzed using a 2-way ANOVA to test the interaction effect of GD and PWD. * *p* < 0.05 by Student’s T-test comparing groups stratified by PWD. Abbreviations: RC-NF: recommended choline (1g/kg) gestational diet weaned to normal fat (16%Kcal) post-weaning diet; HC-NF: high choline (2.5 g/kg) gestational diet weaned to normal fat (16% Kcal) post-weaning diet; RC-HF: recommended choline (1 g/kg) gestational diet weaned to high fat (45%Kcal) post-weaning diet; HC-HF: high choline (2.5 g/kg) gestational diet weaned to high fat (45%Kcal) post-weaning diet.

**Table 4:** Hepatic fatty acid ratios in adult male Wistar rat offspring ^1^.

	RC-NF	HC-NF	RC-HF	HC-HF	*P _GD_*	*P _PWD_*	P _GD*PWD_
C 16:1 *n*-7/C16:0	0.08 ± 0.008	0.13 ± 0.013*	0.049 ± 0.006	0.037 ± 0.003	0.07	0.01	0.01
C18:1 *n*-9/C18:0	0.73 ± 0.07	0.84 ± 0.09	1.63 ± 0.24	0.98 ± 0.15 *	0.09	0.01	0.02
DHA/ARA	0.36 ± 0.02	0.36 ± 0.02	1.19 ± 0.12	1.69 ± 0.06 *	0.36	0.33	0.21
DHA/EPA	22.5 ± 3.5	21.9 ± 1.8	20.4 ± 2.5	33.7 ± 6.55	0.12	0.24	0.11
*n*-6/*n*-3	4.28 ± 0.21	4.23 ± 0.22	5.43 ± 0.21	4.52 ± 0.23 **	0.03	0.01	0.05

^1^ Male Wistar offspring were born to dams fed either a recommended choline (RC) or a high choline (HC) gestational diet (GD) then weaned to either a normal fat (NF) or high fat (HF) post-weaning diet (PWD) for 17 weeks. Values are mean ± SEM, *n* = 8–9/group. Data were analyzed using a 2-way ANOVA to test the interaction effect of GD and PWD. * *p* < 0.05, ** *p* < 0.01 by Student’s T-test comparing groups stratified by PWD. Abbreviations: RC-NF: recommended choline (1 g/kg) gestational diet weaned to normal fat (16%Kcal) post-weaning diet; HC-NF: high choline (2.5 g/kg) gestational diet weaned to normal fat (16% Kcal) post-weaning diet; RC-HF: recommended choline (1 g/kg) gestational diet weaned to high fat (45%Kcal) post-weaning diet; HC-HF: high choline (2.5 g/kg) gestational diet weaned to high fat (45%Kcal) post-weaning diet; DHA: docosahexaenoic acid; ARA: arachidonic acid; EPA: eicosapentaenoic acid; *n*-6: omega-6 polyunsaturated fatty acids; *n*-3: omega-3 polyunsaturated fatty acids.

## Data Availability

All data presented in this study are available in this article and Supplementary Material. Follow-up analyses in this paper were done using a subset of data extrapolated from a recently published research article: Hammoud, R.; Pannia, E.; Kubant, R.; Liao, C.S.; Ho, M.; Yang, N.V.; Chatterjee, D.; Caudill, M.A.; Malysheva, O.V.; Pausova, Z.; Anderson, G.H. 2020. Maternal Choline Intake Programs Hypothalamic Energy Regulation and Later-Life Phenotype of Male Wistar Rat Offspring. doi:10.1002/mnfr.201901178.

## Supplementary Materials

The following are available online at https://www.mdpi.com/article/10.3390/nu13051438/s1. Table S1: Gestational and post-weaning diet composition. Table S2: Fatty acid composition of the post-weaning diets. Table S3: Litter size, body weight, and plasma insulin and leptin concentration of pups at birth. Table S4: Body weight and weekly food intake of dams fed either RC or HC diets during gestation.
