# Supplementary material for: High Choline Intake during Pregnancy Reduces Characteristics of the Metabolic Syndrome in Male Wistar Rat Offspring Fed a High Fat But Not a Normal Fat Post-Weaning Diet
Source: Nutrients. 2021 Apr 24;13(5):1438. doi: 10.3390/nu13051438 (PMC8145686; doi:10.3390/nu13051438)
Supplement: Supplementary file 1 [file nutrients-13-01438-s001.zip › nutrients-1176816-SI.pdf]

nutrients MDPI

Table S1: Gestational and post-weaning diet composition

[1] Mineral Mix (S10022G); [2] Vitamin Mix (V10037). Abbreviations: RC: recommended choline gestational diet; HC: high choline gestational diet; NF: normal fat post-weaning diet; HF: high fat post-weaning diet.

	Gestational Diets		Post-weaning Diets	
	**RC** (D10012G)	**HC** (D17011402)	**NF** (D10012G)	**HF** (D07101303R)
**Composition**	**gm% (Kcal%)**			
Protein	20 (20)	20 (20)	20 (20)	24(20)
Carbohydrate	64 (64)	64 (64)	64 (64)	41 (35)
Fat	7 (16)	7 (16)	7 (16)	24 (45)
Energy Density	4	4	4	4.8
**Ingredients**	**g/kg (Kcal)**			
Casein	200 (800)	200 (800)	200 (800)	200 (800)
L- Cystine	3 (12)	3 (12)	3 (12)	3 (12)
Lard	0	0	0	130 (1170)
Soybean Oil	70 (630)	70 (630)	70 (630)	70 (630)
t-Butylhydroquinone	0.014 (0)	0.014 (0)	0.014 (0)	0.014 (0)
Corn Starch	397 (1590)	397 (1590)	397 (1590)	105 (420)
Maltodextrin	132 (528)	132 (528)	132 (528)	132 (528)
Sucrose	100(400)	100(400)	100(400)	100(400)
Cellulose	50 (0)	50 (0)	50 (0)	50 (0)
Mineral mix [1]	35 (0)	35 (0)	35 (0)	35 (0)
Vitamin mix[2]	10 (40)	10 (40)	10 (40)	10 (40)
Folic Acid	0.002 (0)	0.002 (0)	0.002 (0)	0.0024 (0)
Choline Bitartrate	2.5 (0)	6.25 (0)	2.5 (0)	3 (0)
Free Choline	1 (0)	2.5 (0)	1 (0)	1.2 (0)

Table S2: Fatty Acid Composition of the post-weaning diets[1].

	**Normal Fat** (D10012G)	**High Fat** (D07101303R)
**Fat Source**	**gm**	
Soybean Oil	70	70
Lard	0	130
**Fatty Acids**		
Acetic Acid (C2:0)	0	0
Butyric Acid (C4:0)	0	0
Caproic Acid (C6:0)	0	0
Caprylic Acid (C8:0)	0	0
Capric Acid (C10:0)	0	0
Lauric Acid (C12:0)	0	0.1
Myristic Acid (C14:0)	0.1	1.6
Palmitic Acid (C16:0)	7.2	32.3
Stearic Acid (C18:0)	2.7	16.5
Arachidic Acid (C20:0)	0.2	0.5
Behenic Acid (C22:0)	0.2	0.2
Lignoceric Acid (C24:0)	0.1	0.1
Palmitoleic Acid (C16:1n-7)	0.1	1.9
Vaccenic Acid (C18:1n-7)	0	0
Oleic Acid (C18:1n-9)	16.1	58.8
Eicosenoic Acid (C20:1n-9)	0.2	1
Linoleic Acid (C18:2n-6)	36.2	67.9
Gamma Linolenic Acid (C18:3n-6)	0	0
Eicosadienoic Acid (C20:2n-6)	0	1.1
Dihomo-γ-linolenic acid (C20:3n-6)	0	0.2
Arachidonic Acid (C20:4n-6)	0	0.4
Adrenic Acid (C22:4n-6)	0	0
Docosapentainoic Acid (C22:5n-6)	0	0.1
Alpha-linolenic acid (ALA, C18:3n-3)	5.2	6.9
Eicosatrienoic acid (ETE, C20:3n-3)	0	0
Eicosapentaenoic acid (EPA, C20:5n-3)	0	0
Docosapentaenoic Acid (DPA, C22:5n-3)	0	0.1
Docosahexaenoic acid (DHA, C22:6n-3)	0	0
**Total**	68.3	190.1
Total Saturated, *gm (%)*	10.6 (15.5)	51.9 (27.3)
Total Monounsaturated, *gm (%)*	16.3 (23.9)	61.7 (32.4)
Total Polyunsaturated, *gm (%)*	41.4 (60.6)	76.5 (40.3)
Total n-3, *gm*	5.2	7
Total n-6, *gm*	36.2	68.5
n-6/n-3 ratio	7	9.8

Table S3: Litter size, body weight, and plasma insulin and leptin concentration of pups at birth

	RC	HC	
**Litter Size**	13 ± 1	13 ± 1	0.22
**Pup Body Weight (g)**			
Female Pups	6.4 ± 0.1	6.7 ± 0.2	0.23
Male Pups	6.9 ± 0.2	7.0 ± 0.2	0.54
**Plasma Leptin (ng/ml)**	0.88 ± 0.14	0.44 ± 0.05**	***0.008***
**Plasma Insulin (ng/ml)**	0.3 ± 0.08	0.3 ± 0.06	0.09

Values are Mean±SEM, n=10-12/group. *P<0.05, **P<0.01 by student's T-test.

Abbreviations: RC: AIN-93G diet with recommended choline gestational diet (1g/kg choline from choline bitartrate); HC: high choline gestational diet (2.5 g/kg choline from choline bitartrate).

Table S4: Body weight and weekly food intake of dams fed either RC or HC diets during gestation.

	RC	HC	P-value
**Body Weight (g)**			
Arrival	229.9 ± 4.4	233.0 ± 3.4	0.57
Birth	345.4 ± 8.2	335.3 ± 6.1	0.32
**Food Intake (g)**			
Pregnancy			
Week 1	151.9 ± 5.3	150.7 ± 4.1	0.36
Week 2	191.5 ± 5.5	181.5 ± 4.8	0.06
Week 3	180.6 ± 6.5	173.3 ± 6.9	0.19
Total (g)	524.0 ± 15.1	505.6 ± 13.4	0.09
Lactation			
Week 1	227.0 ± 10.7	208.9 ± 11.1	0.34
Week 2	309.0 ± 9.7	300.4 ± 6.0	0.37
Week 3	423.0 ± 13.6	412.0 ± 8.5	0.44
Total (g)	959.7 ± 31.8	926.3 ± 20.6	0.35

Values are Mean±SEM, n=10-12/group. P<0.05 by student's T-test. Abbreviations: RC: AIN-93G diet with recommended choline gestational diet (1g/kg choline from choline bitartrate); HC: high choline gestational diet (2.5 g/kg choline from choline bitartrate).
